# Serum carnosinase 1, an early indicator for incident microalbuminuria in type 1 diabetes

**DOI:** 10.1007/s40200-024-01422-6

**Published:** 2024-04-03

**Authors:** Jiedong Qiu, Benito A. Yard, Bernhard K. Krämer, Henk J. G. Bilo, Aimo Kannt, Harry van Goor, Peter R. van Dijk

**Affiliations:** 1 5Th Medical Department, University Hospital Mannheim, Heidelberg University, E68167 Mannheim, Germany; 2 Department of Pathology and Medical Biology, University Medical Centre Groningen and University of Groningen, NL-9713 GZ Groningen, the Netherlands; 3 Department of Internal Medicine, University Medical Centre Groningen and University of Groningen, NL-9713 GZ Groningen, the Netherlands; 4 Isala Diabetes Centre, NL-8025 AB Zwolle, the Netherlands; 5 Fraunhofer Institute for Translational Medicine and Pharmacology, E60596 Frankfurt, Germany

**Keywords:** Diabetic nephropathy, Oxidative stress, Carnosine, Histidine-dipeptidase

## Abstract

**Aims:**

Carnosinase (CN1) polymorphisms have been linked to diabetic kidney disease (DKD), as CN1 degrades dipeptides which scavenge oxidative metabolites and prevent the formation of advanced glycation end-products. In this work, we studied the association between serum CN1, the systemic redox status and long-term renal outcome in type 1 diabetes.

**Methods:**

Serum CN1 was measured in a prospective type 1 diabetes cohort (*n* = 218) with a 16-year follow-up. A total of 218 patients treated at the Diabetes Outpatient Clinic of the Weezenlanden Hospital (nowadays Isala Hospital, Zwolle, The Netherlands) were included in this analysis. We assessed whether serum CN1 was associated with renal function and development of DKD as well as other diabetic complications.

**Results:**

At baseline, age, systemic redox status and N-terminal pro brain-natriuretic peptide (NT-proBNP) were associated with serum CN1 concentration (*p* < 0.05). During follow-up, CN1 concentration in the middle tertile was associated with less incident microalbuminuria (odds ratio = 0.194, 95% C.I.: 0.049—0.772, *p* = 0.02) after adjustment for age, systemic redox status, NT-proBNP and sex.

**Discussion:**

Serum CN1 could predict incident microalbuminuria and may be used as a novel parameter to identify patients at risk for DKD.

## Introduction

Carnosinase (CN1) is a serum enzyme degrading histidine-containing dipeptides, i.e. carnosine, anserine and homocarnosine. Because of the beneficial properties of these dipeptides including anti-oxidative effects, increased serum CN1 concentrations have been associated with a deleterious redox state and disease conditions [1–3]. Genetic CN1variants that are associated with low serum CN1 concentrations are more frequently found in patients with type 2 diabetes and without renal function impairment, suggesting a protective effect of lower CN1 concentrations [4–7].

Beside the genetic polymorphism, several factors determine the serum CN1 concentration and activity. Under hyperglycemic conditions, increased N-glycosylation stimulates the secretion of CN1 into the blood flow and moreover it increases CN1 activity itself [8]. Similarly, reactive metabolites, such as methylglyoxal and nitric oxide, can upregulate CN1 activity by carbonylation and S-nitrosylation [9]. On the contrary, S-cysteinylation inhibits serum CN1 [10].

For type 1 diabetes mellitus, no data is available. Therefore, we studied the association between serum CN1 and the systemic redox status as well as development of DKD during a long-term follow-up.

## Subjects, materials and methods

### Cohort

Details of the prospective observational cohort have been described previously [11]. The aim of the observational study was to investigate oxidative stress and quality of life in patients with prospective T1DM [12]. From January 1995 to January 1996, patients with type 1 diabetes were recruited at the outpatient clinic of the Weezenlanden Hospital (now Isala Hospital, Zwolle, the Netherlands). The only inclusion criterion for the study was the presence of type 1 diabetes. After giving their informed consent, participants were included without applying any exclusion criteria. Type 1 diabetes was defined as insulin therapy within 6 months after the manifestation and an age below 30 years, or the absence of C-peptide secretion. For the current study, baseline characteristics were obtained in 2002. The follow-up period was until 2018. In 2018, follow-up data regarding complications and vital status was gathered for the analysis. Initially, 293 patients were recruited from 1995 to 1996. In the period from 1996 to 2002, a total of 32 patients dropped out of the study. Reasons were moving out of the area or referral to another physician (*n* = 12), unknown (*n* = 10), lack of interest (*n* = 6), death (*n* = 2), and incorrect diagnosis of T1DM (*n* = 2). For the current study, data from the 261 patients who participated from 2002 onwards were available. Of these 261 patients, sufficient serum could not be obtained from the 2002 clinical visit of 43 patients. Therefore, we measured serum CN1 in a total of 218 patients with T1DM in this analysis.

We investigated cross-sectional associations between baseline serum CN1 concentrations and diabetic micro- / macrovascular complications as well as clinical parameters. Incidence of complications and mortality rate were recorded prospectively until 2018 for 16 years and employed to assess the prognostic value of serum CN1 concentrations for the development of micro- and macrovascular complications.

Macrovascular complications were defined as angina pectoris (AP), peripheral artery disease (PAD), MI, percutaneous transluminal coronary angioplasty (PTCA), coronary artery bypass grafting (CABG), cerebrovascular accident (CVA) or transient ischemic attack (TIA). Microvascular complications were defined as diabetic retinopathy, albuminuria (both microalbuminuria and macroalbuminuria) and diabetic peripheral neuropathy. Microalbuminuria was defined as 20–200mg/l albumin or an albumin-creatinine ratio between 2.5 and 25mg/mmol (22–221 mg/g) in men and 3.5 and 35mg/mmol (31–310mg/g) in women. Macroalbuminuria was defined as > 200mg/l albumin or a albumin-creatinine ratio greater than 25 mg/mmol and 35 mg/mmol for men and women, respectively. An ophthalmologist determined the presence of diabetic retinopathy biannually. Foot sensibility was tested with 5.07 Semmes–Weinstein monofilaments. Neuropathy was defined as two or more errors in a test of three, affecting at least one foot.

### CN1 measurements

Serum CN1 was measured using a standardized sandwich ELISA, as previously described [13, 14]. Briefly, high-absorbent microtiter flat-bottom plates (#655061, Greiner, Germany) were incubated with 100µl 10µg/ml goat polyclonal anti human carnosine-dipeptidase 1 antibody (#AF2489, R&D, Germany) overnight at 4°C. After washing, unspecific binding was inhibited by incubation with 0.05% milk powder in PBS for 40 min. Serum samples were then added in a dilution of 1/100. After 1h incubation and extensive washing, a rabbit polyclonal CN1 antibody (HPA008933, Sigma, Germany) was diluted 1/600 in PBS and 100µl of the solution was added to each well for 1h. As secondary antibody, a goat anti-rabbit IgG HRP-conjugated antibody (G21234, Thermo Fisher Scientific, USA) in a dilution of 1/1000 with PBS was incubated for 30min. For color development, soluble BM blue peroxidase substrate (#11484281001, Roche, Germany) was employed. The reaction was stopped after 20 min by the addition of 50 µl 1M sulfuric acid (#339741, Sigma, Germany). The quantity was measured on a Tecan Spark 20M plate reader (Tecan, Switzerland) at the wavelength of 450 nm against the reference at 690 nm. A five-parameter logistic regression analysis based on a standard dilution series of recombinant CN1 protein (#10077-H08H, SinoBiological, China) using Prism 8 (GraphPad Software, USA) was performed and serum concentrations of samples interpolated.

### Statistics

SPSS Statistics 26 (Windows version, IBM, USA) was used for all statistical analyses. A two-sided alpha level of 0.05 was considered significant.

Descriptives of the study cohort are shown as the mean with standard deviation (SD) for normally distributed variables and the median with the interquartile range [25th-75th percentile] for non-normally distributed variables. Hereby, normality was checked with normality plots for each variable. Absolute number of patients (% of all patients) for each serum CN1 tertile is shown for categorical variables. One-way ANOVA test was used to analyse different CN1 tertiles on continuous parameters. Assumption of approximately normal distribution in each tertile was tested using normality plots. Homogeneity of variances was tested using the Levene's test and Welch-correction applied if necessary. In case of a non-normal distribution, non-parametric Kruskal–Wallis-test was conducted instead. Chi-square test on categorical parameters and Fisher’s Exact test for p-value for categorical parameters with low number of events at baseline was performed to analyse different CN1 tertiles.

Event-free time of CN1 tertiles from 2002 to 2018 was calculated using Kaplan–Meier estimators and tested with log-rank test. For microalbuminuria, logistic regression for the incidence during the follow-up was performed additionally for adjustment. Cox regression was rejected since the proportional hazard assumption was violated as seen on the survival plot.

A sensitivity analysis for smoking status was performed where the same analyses were performed separately in smokers and non-smokers.

## Results

Baseline characteristics of participants are presented in Table 1. At baseline, mean age of the cohort was 45.2 (11.6) years, 56% were male, mean diabetes duration was 23.6 (10.3) years and HbA1c 59.8 (11.4) mmol/mol or 7.6 (1.0) %, and 12.8% of the patients had microalbuminuria and 3.2% macroalbuminuria with a mean eGFR of 125.7 (25.3) ml/min/1.73m^2^.

**Table 1:** Baseline characteristics

	Data available for *N*	Whole cohort	Tertiles of serum CN1 concentration		
			Low (.006-.065 µg/ml) *n* = 72	Middle (.065-.251 µg/ml) *n* = 73	High (.252–103.35 µg/ml) *n* = 73
Demographics					
Age (years)*	**218**	**45.2 (11.6)**	**45.3 (6.3)**	**42.5 (9.1)**	**47.7 (16.4)**
Diabetes duration (years)	217	23.6 (10.3)	22.4 (9.1)	23.7 (9.4)	24.7 (12.2)
Male sex (n)	218	123 (56.4%)	40 (55.6%)	43 (58.9%)	40 (54.8%)
BMI (kg/m^2^)	191	26.4 (4.0)	26.0 (4.00)	26.8 (3.7)	26.5 (4.6)
Current smoker (yes)	180	50 (27.8%)	22 (33.8%)	12 (19.0%)	16 (30.8%)
Systolic blood pressure (mmHg)	195	130.8 (17.8)	131.0 (17.8)	130.1 (16.8)	131.5 (19.3)
Diastolic blood pressure (mmHg)	195	77.3 (10.1)	77.8 (9.5)	76.7 (10.6)	77.4 (10.2)
Mode of insulin administration: MDI (*n*)	218	124 (56.9%)	41 (56.9%)	39 (53.4%)	44 (60.3%)
Mode of insulin administration: CSII (*n*)	218	94 (43.1%)	31 (43.1%)	34 (46.6%)	29 (39.7%)
Complications					
Microvascular complications					
Retinopathy (*n*)	218	82 (37.6%)	26 (36.1%)	32 (43.8%)	24 (32.9%)
Neuropathy (*n*)	218	32 (14.7%)	9 (12.5%)	11 (15.1%)	12 (16.4%)
Micro-albuminuria (*n*)	218	28 (12.8%)	9 (12.5%)	8 (11.0%)	11 (15.1%)
Macro-albuminuria (*n*)	218	7 (3.2%)	2 (2.8%)	1 (1.4%)	4 (5.5%)
Macrovascular events					
AP (*n*)	218	4 (1.8%)	3 (4.2%)	0 (0%)	1 (1.4%)
MI (*n*)	218	3 (1.4%)	1 (1.4%)	0 (0%)	2 (2.7%)
PTCA (*n*)	218	3 (1.4%)	1 (1.4%)	0 (0%)	2 (2.7%)
PAD (*n*)	218	5 (2.3%)	1 (1.4%)	2 (2.7%)	2 (2.7%)
CABG (*n*)	218	3 (1.4%)	0 (0.0%)	0 (1.4%)	3 (4.1%)
TIA (*n*)	218	2 (0.9%)	0 (0.0%)	1 (1.4%)	1 (1.4%)
CVA (*n*)	218	4 (1.8%)	1 (1.4%)	2 (2.7%)	1 (1.4%)
Laboratory measurements					
HbA1c (%)	212	7.6 (1.0)	7.8 (0.9)	7.6 (1.1)	7.5 (1.1)
HbA1c (mmol/mol)	212	59.8 (11.4)	61.7 (10.3)	59.7 (12.1)	58.0 (11.6)
Creatinine (µmol/L)	209	85.5 (13.1)	84.3 (10.0)	86.1 (11.1)	86.0 (17.2)
eGFR (ml/min/1.73m^2^)	178	107.6 (28.6)	104.6 (22.8)	110.0 (26.1)	108.6 (37.4)
Total cholesterol (mmol/L)	212	4.6 (1.0)	4.6 (1.0)	4.6 (1.1)	4.7 (0.9)
HDL cholesterol (mmol/L)	211	1.5 [1.2, 1.9]	1.5 [1.3, 1.9]	1.3 [1.2, 1.9]	1.5 [1.2, 1.9]
Total cholesterol:HDL ratio	210	3.1 [2.5, 3.8]	3.0 [2.4, 3.8]	3.1 [2.5, 4.1]	3.1 [2.5, 3.6]
LDL cholesterol (mmol/L)	211	2.6 (0.9)	2.6 (1.0)	2.6 (0.9)	2.6 (0.8)
Triglycerides (mmol/L)	211	0.9 [0.7, 1.4]	0.9 [0.7, 1.3]	0.9 [0.6, 1.4]	0.9 [0.7, 1.4]
C-reactive protein (mg/L)	206	2.0 [1.00, 3.00]	1.5 [1.0, 3.0]	2.0 [1.0, 4.0]	2.0 [1.0, 4.0]
R-SH (µM)***	**215**	**285 [257, 304]**	**261 [245, 286]**	**286 [264, 302]**	**300 [280, 324]**
NT-proBNP (pmol/L)*	**201**	**40.5 [16.2, 86.2]**	**57.4 [20.5, 96.8]**	**50.3 [16.2, 117.1]**	**31.1 [12.8, 59.2]**
Albumin (g/l)	206	42.2 (2.6)	42.3 (2.8)	42.4 (3.0)	42.1 (2.2)

*for a *p*-value < 0.05 and *** for a *p-*value < 0.001

*BMI *body mass index, *CABG *coronary artery bypass grafting, *CSII *continuous subcutaneous insulin infusion, *CVA *cerebral vascular event, *eGFR *estimated glomerular filtration rate, *HbA1c *glycated hemoglobin A1c, *HDL *high-density lipoprotein, *LDL *low-density lipoprotein, *MDI *multiple daily injections, *MDRD *Modification of diet in renal disease, *MI *myocardial infarction, *NA *not applicable, *PTCA *percutaneous transluminal coronary angioplasty, *R-SH *total free thiol groups, *TIA *transient ischemic attack

While at baseline 37.6% (*N* = 82 of 218 patients) already had diabetic retinopathy, during the 16-year follow-up period this increased to 62.4% and there was no difference between CN1 tertiles (Mantel-Cox *χ*^*2*^ (2) = 2.87, *p* = 0.238). Similarly, for the prevalence of diabetic neuropathy, which increased from 14.7 to 31.2% (32 to 68 of 218 patients) during the follow-up, no difference was found for CN1 tertiles (Mantel-Cox *χ*^*2*^ (2) = 1.08, *p* = 0.583).

**Fig. 1:**
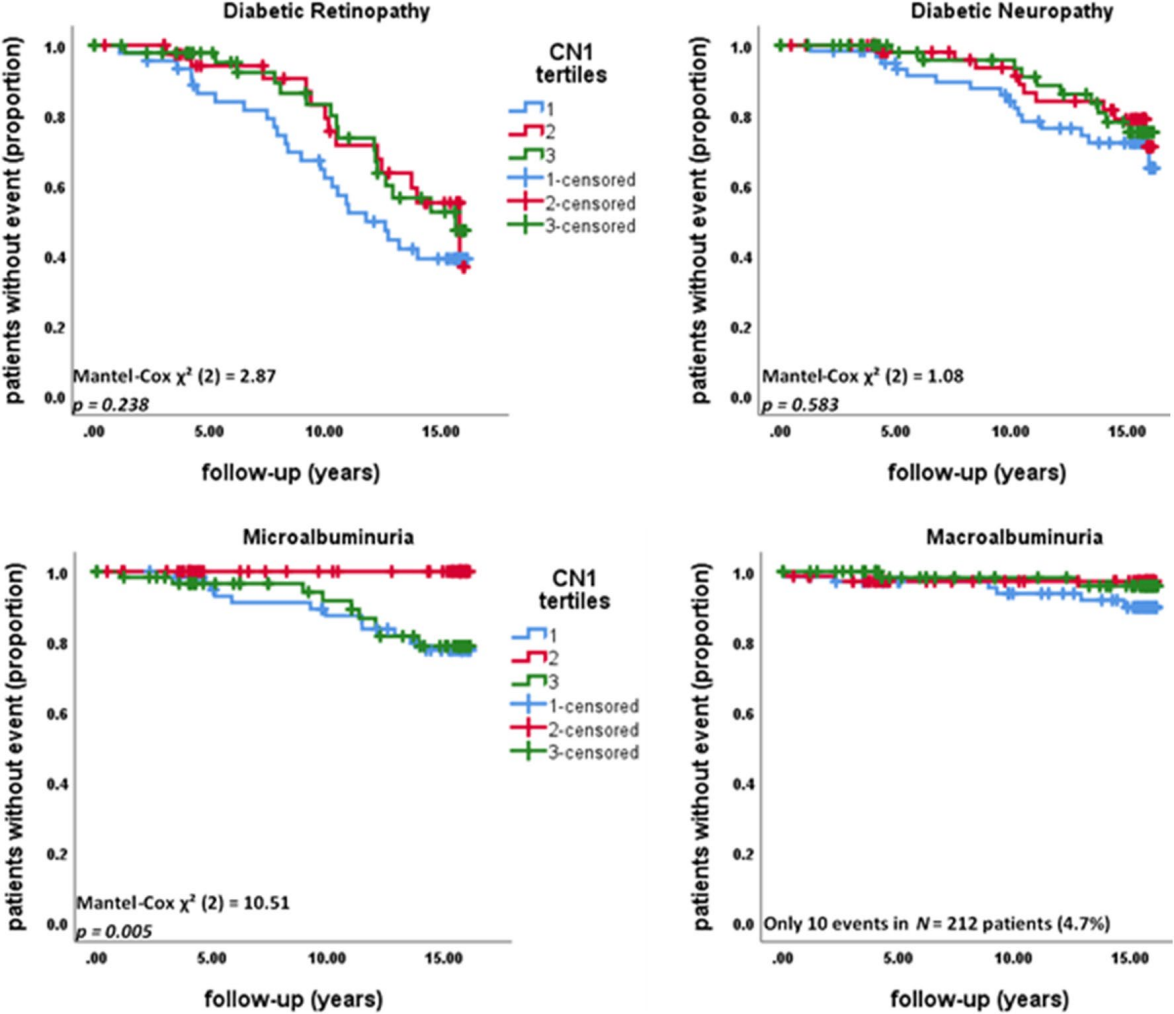
16-year follow-up incidence in diabetic retinopathy, diabetic neuropathy, microalbuminuria and macroalbuminuria are depicted as Kaplan–Meier curves. 218 Patients with type 1 diabetes were followed from 2002 to 2018. We extracted the incidence of the microvascular complications. Patients with the selected complication at baseline or patients lost during the follow-up were censored. Event-free (without the selected complication) time was calculated using Kaplan–Meier estimators stratified according to the CN1 tertiles and tested with log-rank test

During follow-up, the prevalence of microalbuminuria increased from 12.8 to 22.4% (28 to 49 of the 218 patients). Patients in the middle CN1 tertile without microalbuminuria at baseline were less likely to develop microalbuminuria during the 16-year follow-up (Mantel-Cox *χ*^*2*^ (2) = 10.5, *p* = 0.005). Whereas in the lowest CN1 tertile 19.4% (*N* = 12 of 63 patients) and in the highest CN1 tertile 14.5% (*N* = 9 of 62 patients) developed microalbuminuria during the follow-up, in the middle tertile none of the 65 patients developed microalbuminuria (Fig. 1). Only 4.8% of patients (*N* = 10 of 210) developed macroalbuminuria.

The association between CN1 tertile and microalbuminuria was further assessed in a multivariable binary logistic regression, adjusting for age, serum free thiols (R-SH) and NT-proBNP, parameters, which significantly differed between CN1 tertiles at baseline, and for sex. The created model showed a significant association with development of microalbuminuria during follow-up (Χ^2^ (5) = 24.87, *p* < 0.001), i.e. being one year older increased the odds by a factor of 1.04 (95% *C.I.*: 1.01 – 1.08, *p* = 0.01) and having a CN1 concentration in the second tertile decreased the odds ratio to 0.66 (95% *C.I.:* 0.12 – 0.99, *p* = 0.047).

Furthermore, we performed a sensitivity analysis to test whether smoking status has an effect on the prediction of microalbuminuria by serum CN1 levels. The association could be reproduced in non-smokers (*p* = 0.02), while it was not significant in smokers (*p* = 0.25), in which the analysis was underpowered due to less events (4 and 1 in the highest and lowest tertile, *N* = 44 in total) in the follow-up despite a higher risk than in non-smokers. Also here, no new case of microalbuminuria was detected in patients within the middle CN1 tertile.

There was no difference in macrovascular complications or all-cause mortality between CN1 tertiles during follow-up (Mantel-Cox *χ*^*2*^ (2) = 0.99 and 2.78, *p* > 0.05).

## Discussion

In our prospective type 1 diabetes cohort, serum CN1 at baseline was associated with age, systemic redox status and NT-proBNP, while patients with a moderate CN1 concentration at baseline seemed to be protected from incident albuminuria as compared to the other CN1 tertiles (14.5 – 19.4%) during the 16 years of follow-up.

In this work, systemic redox status was reflected by R-SH, which were associated with NT-proBNP, suggesting a link between redox status and the increased risk for heart failure in patients with diabetes [11]. In contrast to CN1, R-SH was not associated with diabetic complications [11], indicating a better prognostic value of CN1.

The association between CN1 and microalbuminuria was not seen at baseline, but during the follow-up. This may not only be due to the small number of patients having microalbuminuria at baseline, but also due to the fact that some CN1 is lost with microalbuminuria.

Although in type 2 diabetes the association of DKD with genetic variants of the CN1 gene has been shown by genetic studies [4], its role in type 1 diabetes has been controversial [15, 16]. This might be explained by fundamental differences in insulin metabolism between both diabetes types. Since another benefit of carnosine has been shown in its ability to increase serum insulin concentrations and thus to reduce serum glucose levels [1, 2], CN1 may influence hyperglycemia in type 1 diabetes to a lesser extent than in type 2 diabetes. In line with this hypothesis, HbA1c was not associated with CN1 in this study. Furthermore, serum CN1 has not yet been measured in a cohort with long-term follow-up and may be influenced by several factors including age and sex [17]. In concordance, serum CN1 was associated with age, redox status and NT-proBNP in this study.

Since in the middle tertile, no case of incident microalbuminuria was detected despite a considerable tertile size of *N* = 65 and an incidence of 19.4 and 14.5% in the other tertiles (12 and 9 cases of *N* = 62), serum CN1 may have potential as a prognostic marker for early renal filtration barrier impairment. More studies with larger cohorts are needed to confirm this finding and to estimate its prognostic value.

According to the mitohormesis theory [18], both increased and decreased redox level have a negative impact on physiological functions. This may help understand the bell-shaped relation between CN1 and incident microalbuminuria during the follow-up.

Unexpectedly, R-SH was positively associated with CN1, while NT-proBNP was inversely associated with CN1. Although those relations were highly significant and thus corrected for in the multivariable analysis, we had no good explaination for it. Similarly, as the bardoxolone trial showed that anti-inflammatory agents may lead to increased cardiovascular risk in patients with high NT-proBNP, CN1 polymorsphism has been associated with cardiovascular risk [16]. Whether and how CN1 may affect the cardiovascular system has to be assessed in further studies.

Major limitations of this study include the relatively young age of the patients at baseline, only mild renal impairment during the follow-up with no incidence of renal failure or death and low diversity of the Dutch study population. Given the known discrepancies in renal risk across different ethnic groups [19, 20] and the limited diversity in our Dutch study population, caution is advised when extrapolating these findings to other ethnicities.

In summary, by measuring serum CN1 directly we have shown that both increased and decreased serum CN1 was associated with incident microalbuminuria whereas patients with moderate CN1 concentrations were protected. This parameter might have a merit as a prognostic marker and should be assessed in larger cohorts with harder renal endpoints. Finally, we provided hereby the first evidence that serum CN1 may be associated with DKD in patients with type 1 diabetes.

## Data Availability

Data is available upon reasonable request.
